# Synthesis of Isotactic-*block*-Syndiotactic Poly(methyl Methacrylate) via Stereospecific Living Anionic Polymerizations in Combination with Metal-Halogen Exchange, Halogenation, and Click Reactions

**DOI:** 10.3390/polym9120723

**Published:** 2017-12-16

**Authors:** Naoya Usuki, Kotaro Satoh, Masami Kamigaito

**Affiliations:** 1 Department of Molecular and Macromolecular Chemistry, Graduate School of Engineering, Nagoya University, Furo-cho, Chikusa-ku, Nagoya 464-8603, Japan; usuki@chiral.apchem.nagoya-u.ac.jp; 2 Precursory Research for Embryonic Science and Technology, Japan Science and Technology Agency, 4-1-8 Honcho, Kawaguchi, Saitama 332-0012, Japan

**Keywords:** living polymerization, stereospecific polymerization, anionic polymerization, methyl methacrylate, PMMA, stereoblock polymer, stereocomplex, click reaction, isotactic polymer, syndiotactic polymer

## Abstract

Isotactic (*it*-) and syndiotactic (*st*-) poly(methyl methacrylate)s (PMMAs) form unique crystalline stereocomplexes, which are attractive from both fundamental and application viewpoints. This study is directed at the efficient synthesis of *it*- and *st*-stereoblock (*it*-*b*-*st*-) PMMAs via stereospecific living anionic polymerizations in combination with metal-halogen exchange, halogenation, and click reactions. The azide-capped *it*-PMMA was prepared by living anionic polymerization of MMA, which was initiated with *t*-BuMgBr in toluene at –78 °C, and was followed by termination using CCl_4_ as the halogenating agent in the presence of a strong Lewis base and subsequent azidation with NaN_3_. The alkyne-capped *st*-PMMA was obtained by living anionic polymerization of MMA, which was initiated via an in situ metal-halogen exchange reaction between 1,1-diphenylhexyl lithium and an α-bromoester bearing a pendent silyl-protected alkyne group. Finally, copper-catalyzed azide-alkyne cycloaddition (CuAAC) between these complimentary pairs of polymers resulted in a high yield of *it*-*b*-*st*-PMMAs, with controlled molecular weights and narrow molecular weight distributions. The stereocomplexation was evaluated in CH_3_CN and was affected by the block lengths and ratios.

## 1. Introduction

Recent advances in controlled polymerizations, including living and stereospecific polymerizations, as well as highly efficient reactions, such as click reactions, enable the synthesis of various architecturally controlled polymers [1]. In addition, an alternative and more efficient synthetic route for polymer structures can be created by these developments, which is the case in the synthesis of organic molecules, such as medicines and natural products due to the development of new reactions.

Typical stereoregular poly(methyl methacrylate)s (PMMAs), i.e., isotactic (*it*-) and syndiotactic (*st*-) PMMAs, are known to associate in the solid state and specific solutions to form a crystalline polymer complex, i.e., a stereocomplex, in which inner single or double helixes of the *it*-PMMA chain are surrounded by an outer *st*-PMMA helical chain with a larger radius [2,3,4,5,6,7,8,9,10]. The unique structures and properties of synthetic polymers have attracted attention from both fundamental and application viewpoints [11,12,13,14,15,16,17,18,19]. Furthermore, these stereoregular polymer chains have been incorporated into various well-defined polymers, such as block, star, graft, and end-functionalized polymers, to create novel properties and functions [20,21,22,23,24,25,26,27,28,29,30,31,32,33,34,35,36,37,38,39].

Stereoblock PMMAs that consist of *it*- and *st*-PMMA blocks connected in one linear chain have been prepared via various approaches. Although there have been several reports on the formation of *it*-*b*-*st*-stereoblock PMMAs via anionic polymerization under certain conditions since the 1950s, they are mostly mixtures of *it*- and *st*-PMMAs or are randomly segmented *it*- and *st*-PMMA blocks with uncontrolled block lengths and broad molecular weight distributions (MWDs) [3,40,41,42,43,44,45,46,47,48,49]. The well-defined *it*-*b*-*st*-stereoblock PMMA was first synthesized in 1986 via a sequential living anionic block copolymerization of two monomers, diphenylmethyl and triphenylmethyl methacrylates, which resulted in syndiotactic (*rr* = 84%) and isotactic (*mm* = 91%) sequences, respectively, followed by hydrolysis and methylation of their ester moieties [22]. Later, the direct synthesis of an *it*-*b*-*st*-stereoblock PMMA was achieved via the transformation of the isotactic living anionic polymerization of MMA, initiated with *t*-BuMgBr in toluene at −60 °C (*mm* = 96%) into a syndiotacitc polymerization via the addition of Me_3_Al at a ratio of Al/Mg ≥ 6 at −78 °C (*rr* ≤ 75%) [23]. Another synthetic route to synthesize *it*-*b*-*st*-stereoblock PMMA was reported via a coupling reaction between the alcohol-capped *it*- (*mm* = 97%) and *st*-PMMAs (*rr* = 89%) by sebacoyl dichloride, where the two prepolymers were prepared by living anionic polymerizations of MMA with *t*-BuMgBr and *t*-BuLi/*n*-Bu_3_Al, respectively, in toluene at −78 °C followed by termination with allyliodide and a subsequent hydroboration-oxidation [24,25,26,27]. This approach was further used for the synthesis of uniform *it*-*b*-*st*-stereoblock PMMA with a single molecular weight via fractionation using preparative size-exclusion chromatography (SEC) and supercritical fluid chromatography (SFC). The stereocomplexation behavior was analyzed in detail. However, this coupling reaction statistically results in 50% homo-coupling products (*it*-*b*-*it* and *st*-*b*-*st*), which should be separated from the desired hetero-coupling products by chromatography. A more efficient synthetic approach can be developed by designing polymerization and coupling reactions.

**Scheme 1:**
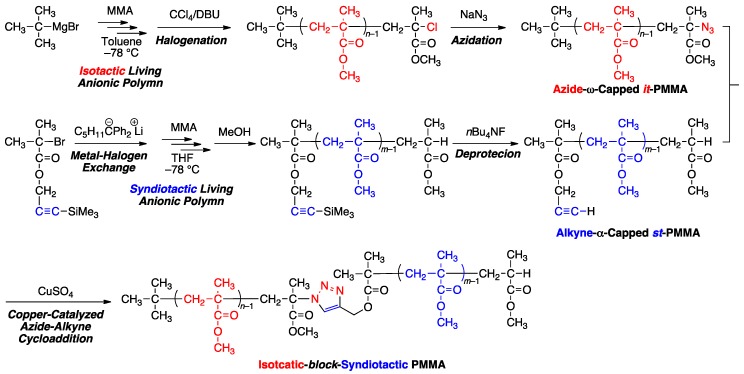
Synthesis of Isotactic-*block*-Syndiotactic Poly(methyl Methacrylate) via Stereospecific Living Anionic Polymerizations in Combination with Metal-Halogen Exchange, Halogenation, and Click Reactions.

We have recently determined that the halogenation of the growing enolate terminal in the isotactic and syndiotactic living anionic polymerization of MMA is efficient and feasible upon the addition of CCl_4_ or CCl_3_Br in the presence of a strong Lewis base, such as 1,8-diazabicyclo[5,4,0]undec-7-ene (DBU) [50]. Furthermore, the halogen terminal of PMMA can be almost quantitatively transformed into an azide terminal via a reaction with sodium azide [33,36]. In addition, an alkyne-capped PMMA can be synthesized by a living anionic polymerization of MMA that is initiated via an in situ metal-halogen exchange reaction between 1,1-diphenylhexyl lithium, i.e., the sacrificial anion source, and an α-haloester bearing a pendent silyl-protected alkyne group, i.e., a precursor of the functionalized initiator [36]. In our previous report, both α-alkyne and ω-azide chain-end functionalizations were used in a single polymer chain of *st*-PMMA to enable the synthesis of cyclic *st*-PMMA. Here, we report a more efficient synthesis of *it*-*b*-*st*-stereoblock PMMA via a copper-catalyzed azide-alkyne cycloaddition (CuAAC) between azide-ω-capped *it*-PMMA and alkyne-α-capped *st*-PMMA, prepared via a stereospecific anionic polymerization (Scheme 1).

## 2. Materials and Methods

### 2.1. Materials

Methyl methacrylate (MMA; TCI, Tokyo, Japan, >99.8%), 1,8-diazabicyclo[5.4.0]undec-7-ene (DBU; TCI, Tokyo, Japan, >98.0%), and CCl_4_ (Kanto, Tokyo, Japan, >99.9%) were distilled from calcium hydride before use. *t*-BuMgBr, diphenylhexyllithium (DPHLi), and 3-(trimethylsilyl)propargyl α-bromoisobutyrate (**1**) were prepared according to the literature [36,50,51,52,53]. Toluene (Kanto, Tokyo, Japan, >99.5%; H_2_O <0.001%) and tetrahydrofuran (THF; Kanto, >99.5%; H_2_O <0.001%) were dried and deoxygenized by passage through columns of Glass Contour Solvent Systems (Glass Contour, Nashua, NH, USA) before use. Tetrabutylammonium fluoride (*n*-Bu_4_NF; TCI, Tokyo, Japan, ca. 1 mol/L in THF), sodium azide (NaN_3_; TCI, Tokyo, Japan, >99.0%), CuSO_4_ (Kanto, Tokyo, Japan, >97.5%), ascorbic acid (TCI, Tokyo, Japan, >99.0%), sodium ascorbate (Kanto, Tokyo, Japan, >98.0%), CH_3_CN (Kanto, Tokyo, Japan, 99.5%), and DMSO (Kanto, Tokyo, Japan, 99.5%) were used as received. CuCl (Aldrich, St. Louis, MO, USA, 99.99%), CuCl_2_ (Aldrich, St. Louis, MO, USA, 99.99%) and 2,2′-bipyridyl (bpy; Aldrich, St. Louis, MO, USA, >99.0%) was used as received and was handled in a glove box (MBRAUN LABmaster sp, Garching, Germany) under a moisture and oxygen-free argon atmosphere (O_2_, <1 ppm).

### 2.2. Synthesis of Azide-ω-Capped it-PMMA via Isotactic Living Anionic Polymerization of MMA Followed by Termination with Halogenation and Subsequent Azidation

The reactions were carried out by syringe techniques under dry argon in a 100 mL baked glass tube equipped with a three-way stopcock. The following is a typical example for *it*-50-PMMA. The polymerization was initiated by adding MMA (5.2 mL, 49 mmol) slowly via a dry syringe into the prechilled initiator solution (31 mL), containing *t*-BuMgBr (0.97 mmol, 3.5 mL, 276 mM in Et_2_O) and toluene (27 mL), at −78 °C. The total volume of the reaction mixture was thus 36 mL. After stirring for 26 h, the THF solution (56 mL) of CCl_4_ (44 mmol, 4.3 mL) and DPHLi (3.4 mmol, 8.4 mL, 400 mM in THF) was added to the reaction mixture. After 1 h, into the reaction mixture was then added DBU (9.7 mmol, 1.5 mL) and the reaction temperature was gradually raised to 0 °C. After additional 24 h, the reaction was quenched by 6.8 mL of argon-bubbled methanol. The quenched solution was diluted with 100 mL of toluene and was washed with diluted hydrochloric acid and water, evaporated to dryness under reduced pressure, and then vacuum-dried to give the product (*M*_n_ = 5000, *M*_w_/*M*_n_ = 1.50) containing low molecular weight residues. The obtained product was dissolved with toluene and reprecipitated into hexane three times to result in the chlorine-capped *it*-PMMA (*M*_n_ = 5100, *M*_w_/*M*_n_ = 1.61, *mm*/*mr*/*rr* = 92/7/1).

The obtained chlorine-capped *it*-PMMA (1.0 g, 0.20 mmol), sodium azide (130 mg, 2.0 mmol), and ascorbic acid (5.3 mg, 0.030 mmol) were dissolved in 20 mL of CH_3_CN/H_2_O (9:1). A catalyst solution of CuCl/CuCl_2_/bpy (0.10/0.067/0.33 M in CH_3_CN/H_2_O (9:1), 1.19 mL) was then added. The flask was immersed in thermostatic oil bath at 40 °C and kept stirred for 18 h. The solution was diluted with 50 mL of toluene, and washed with diluted hydrochloric acid and water. The solution was concentrated and dried to give the azide-ω-ended *it*-PMMA (*M*_n_ = 4800, *M*_w_/*M*_n_ = 1.59).

### 2.3. Synthesis of Alkyne-α-Capped st-PMMA via Syndiotactic Living Anionic Polymerization of MMA Initiated via a Metal-Halogen Exchange Reaction

The reactions were carried out by syringe techniques under dry argon in a 100 mL baked glass tube equipped with a three-way stopcock. The following is a typical example for *st*-50-PMMA. Prior to the polymerization, the functionalized enolate initiator was in situ prepared by adding 6.4 mL of **1** (1.7 mmol, 272 mM in THF) into the THF solution (40 mL) of DPHLi (2.6 mmol) at −100 °C. Into the mixture, MMA (48 mmol, 5.1 mL) was added dropwise, and the total volume of the reaction mixture was thus 52 mL. After stirring for 30 min, the polymerization was terminated with acidic methanol (4.5 mL). The solution was diluted with 100 mL toluene, and was washed with diluted hydrochloric acid and water, evaporated to dryness under reduced pressure, and then vacuum-dried to give the product (*M*_n_ = 6300, *M*_w_/*M*_n_ = 1.11, *mm*/*mr*/*rr* = 1/22/77).

The obtained *st*-PMMA with a protected alkyne (3.4 g, 0.63 mmol) was dissolved in 58 mL of THF. To the solution, 1.3 mL of the THF solution of *n*-Bu_4_NF (1.0 M, 1.3 mmol) was added at 0 °C. After 1 h, the solution was diluted with 30 mL of toluene, washed with diluted hydrochloric acid and water, and evaporated to dryness to the alkyne-α-ended *st*-PMMA (*M*_n_ = 6100, *M*_w_/*M*_n_ = 1.11).

### 2.4. Synthesis of it-b-st-Stereoblock PMMA via Copper-Catalyzed Azide-Alkyne Cycloaddition

The CuAAc reaction was carried out with CuSO_4_ in the presence of sodium ascorbate as the reducing agent in DMSO/H_2_O (98/2) in a baked glass tube equipped with a three-way stopcock. The following is a typical example for *it*-50-*b*-*st*-100-PMMA. The azide-ω-ended *it*-PMMA (*M*_n_ = 4800, *M*_w_/*M*_n_ = 1.59, *F*_n_(N_3_) = 0.74, 247 mg, 0.051 mmol) and alkyne-α-ended *st*-PMMA (*M*_n_ = 12000, *M*_w_/*M*_n_ = 1.11, *F*_n_(C≡C–H) = 1.04, 462 mg, 0.039 mmol) were dissolved in DMSO (1.9 mL). To this solution, 0.04 mL of H_2_O, CuSO_4_ (2.1 mg, 0.013 mmol), and sodium ascorbate (10 mg, 0.051 mmol) were added. The flask was immersed in thermostatic oil bath at 70 °C, and the reaction mixture was allowed to stir for 24 h. The reaction was quenched by dilution with toluene (50 mL). The mixture was washed with diluted hydrochloric acid and water. The solution was evaporated to dryness under reduced pressure in order to give the crude stereoblock PMMA (*M*_n_ = 12400, *M*_w_/*M*_n_ = 1.20). The obtained product was dissolved in toluene and reprecipitated into hexane three times to result in the *it*-50-*b*-*st*-100-PMMA (*M*_n_ = 14000, *M*_w_/*M*_n_ = 1.20).

### 2.5. Measurements

The number-average molecular weight (*M*_n_) and molecular weight distribution (*M*_w_/*M*_n_) of the polymers were measured by size-exclusion chromatography (SEC) using THF at a flow rate 1.0 mL/min at 40 °C on two polystyrene gel columns (Shodex KF-805L (Showa Denko K.K., Tokyo, Japan, pore size: 20–1000 Å; 8.0 mm i.d. × 30 cm)) that were connected to a JASCO PU-2080 precision pump and a JASCO RI-2031 detector (JASCO, Tokyo, Japan). The columns were calibrated against seven standard PMMA samples (Agilent Technologies, Santa Clara, CA, USA; *M*_p_ = 202–1677000, *M*_w_/*M*_n_ = 1.02–1.23). ^1^H and ^13^C NMR spectra were recorded on a JEOL ESC-400 spectrometer (JEOL, Tokyo, Japan) operating at 400 and 100 MHz, respectively. The triad tacticity of the polymer was determined by the peak intensity of carbonyl C=O carbons that were observed at 175–180 ppm in the ^13^C NMR spectrum. The transmittance of polymer solutions was recorded in CH_3_CN using a JASCO V-550 spectrophotometer (JASCO, Tokyo, Japan) (cooling rate = 1 °C/min; wavelength 500 nm). The glass transition (*T*_g_) and melting (*T*_m_) temperatures were recorded on Q200 differential scanning calorimeter (DSC) (TA Instruments Inc., New Castle, DE, USA). *T*_g_ was obtained form the second scan, where the samples were heated to 180 °C at 10 °C/min, equilibrated at 180 °C for 5 min, cooled to 0 °C at 5 °C/min, held at 0 °C for 5 min, and then reheated to 200 °C at 10 °C/min. *T*_m_ was obtained from the first scan, where the samples were heated from 40 to 250 °C at 10 °C/min. The samples for *T*_m_ were obtained by evaporating CH_3_CN from the solution of stereoblock PMMA (50 mg/mL) and dried under vacuo overnight.

## 3. Results and Discussion

### 3.1. Synthesis of Azide-ω-Capped it-PMMA via Isotactic Living Anionic Polymerization of MMA Followed by Termination with Halogenation and Subsequent Azidation

**Figure 1:**
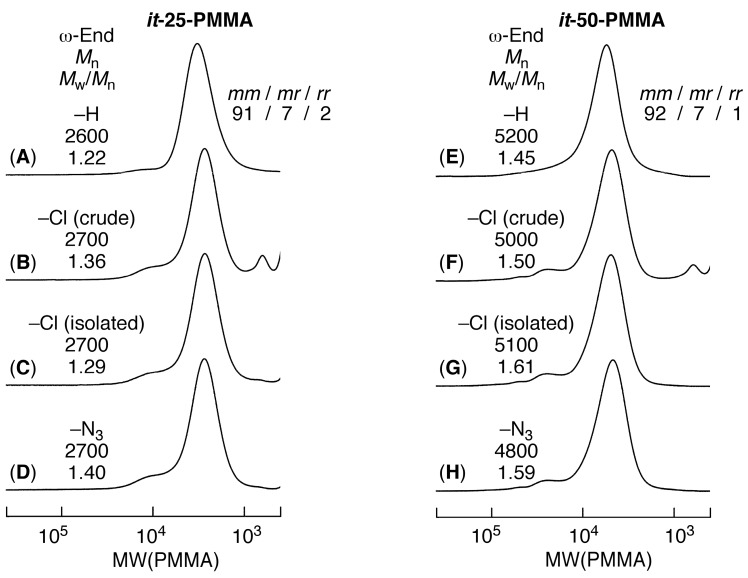
Size-exclusion chromatography (SEC) curves of a series of *it*- poly(methyl methacrylate)s (PMMAs) ((**A**–**D**): *it*-25, (**E**–**H**): *it*-50) obtained in isotactic anionic polymerization of Methyl methacrylate (MMA) with *t*-BuMgBr in toluene at −78 °C and subsequent procedures: (**A**,**E**) Quenching the polymerization ([M]_0_/[*t*-BuMgBr]_0_ = 1350/54 or 1350/27 mM) with acidic methanol; (**B**,**F**) Quenching the polymerization with CCl_4_/DBU ([M]_0_/[*t*-BuMgBr]_0_/[CCl_4_]_add_/[DBU]_add_ = 1350/54/2160/540 or 1350/27/1080/270 mM); (**C**,**G**) Reprecipitations; (**D**,**H**) Azidation with NaN_3_/CuCl/CuCl_2_/bpy in CH_3_CN/H_2_O = 9/1 at 40 °C ([—Cl]_0_/[NaN_3_]_0_/[CuCl]_0_/[CuCl_2_]_0_/[bpy]_0_ = 10/100/6.5/4.3/22 mM).

Isotactic living anionic polymerization of MMA was conducted with *t*-BuMgBr in toluene at −78 °C [51,52] at different molar ratios ([M]_0_/[*t*-BuMgBr]_0_ = 25 and 50) to synthesize *it*-PMMA with different molecular weights. The polymers obtained after quenching the reaction with acidic methanol had controlled molecular weights and narrow MWDs, as shown in the SEC curves (Figure 1A,E). The ^13^C NMR showed isotactic-rich enchainments (*mm*/*mr*/*rr* = 91/7/2 or 92/7/1), as reported [51,52]. The termination with CCl_4_ as the chlorinating agent in the presence of DBU, a strong Lewis base, also resulted in polymers with similar molecular weights and narrow MWDs [50]. Although higher and lower molecular weight fractions were slightly observed, probably being due to high viscosity upon quenching (Figure 1B,F), the SEC curves were nearly unimodal after the reprecipitations (Figure 1C,G).

**Figure 2:**
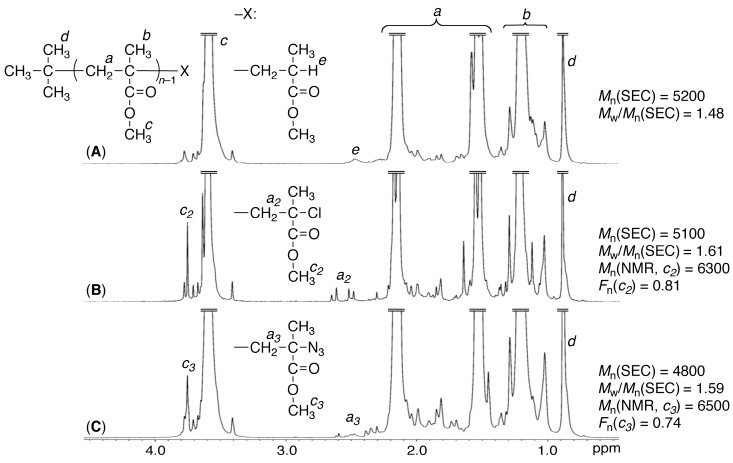
^1^H NMR spectra (in CDCl_3_ at 55 °C) of *it*-50-PMMAs obtained in anionic polymerization of MMA with *t*-BuMgBr in toluene at −78 °C and subsequent procedures: (**A**) Quenching the polymerization ([M]_0_/[*t*-BuMgBr]_0_ = 1350/27) with acidic methanol; (**B**) Quenching the polymerization with CCl_4_/DBU ([M]_0_/[*t*-BuMgBr]_0_/[CCl_4_]_add_/[DBU]_add_ = 1350/27/1080/270) followed by reprecipitations; (**C**) Azidation with NaN_3_/CuCl/CuCl_2_/bpy in CH_3_CN/H_2_O = 9/1 at 40 °C ([–Cl]_0_/[NaN_3_]_0_/[CuCl]_0_/[CuCl_2_]_0_/[bpy]_0_ = 10/100/6.5/4.3/22 mM).

The ^1^H NMR spectrum of the polymers obtained by termination with CCl_4_ shows new peaks, *a*_2_ and *c*_2_, which are assignable to the terminal methyl ester and methylene protons that are adjacent to the chloride chain end, respectively (Figure 2B). The terminal chloride functionality (*F*_n_(Cl)) was determined by the ratio of *M*_n_(SEC), as measured by SEC based on the PMMA calibration to *M*_n_(NMR), which was obtained from the peak intensity ratio of *c*_2_ to the methyl ester protons (*c*) of the repeating MMA units. The obtained *F*_n_(Cl) values were 1.00 and 0.81 for *M*_n_ = 2700 and 5100, respectively. In addition, the *F*_n_(Cl) values that were calculated from the peak area ratio of the ω-end methyl ester (*c*_2_) to the α-end *t*-butyl (*d*) protons were 1.00 and 0.78, respectively. These results indicate that most of the isotactic living chain ends of the PMMAs were capped with chlorine.

The chlorine ω-chain-end polymers were then reacted with NaN_3_ in the presence of copper catalysts in CH_3_CN/H_2_O = 9/1 at 40 °C. The SEC curve of the polymers that were obtained after azidation showed similarly narrow MWDs (Figure 1D,H). The terminal methylene peak (*a*_2_) clearly shifted upfield (*a*_3_) via the transformation from a chloride into an azide terminal (Figure 2C). The chain-end functionality of the azide group (*F*_n_(N_3_)) was similarly calculated by the ratio of *M*_n_(SEC) to *M*_n_(NMR), which was obtained from the peak intensity ratio of the methyl ester protons, *c*_3_ and *c*. The *F*_n_(N_3_) values were 1.00 and 0.74 for *M*_n_ = 2700 and 4800, respectively. In addition, similar values were obtained from the peak area ratio of the ω-end methyl ester (*c*_3_) to the α-end *t*-butyl (*d*) protons were 0.96 and 0.75, respectively. They were close to those for *F*_n_(Cl) and indicated that the chloride terminal was almost quantitatively converted into the azide terminal. These results indicate that the *it*-PMMA with an azide group at the ω-chain end was successfully obtained by isotactic living anionic polymerization of MMA, followed by chlorination for quenching and subsequent azidation.

### 3.2. Synthesis of Alkyne-α-Capped st-PMMA via Syndiotactic Living Anionic Polymerization of MMA Initiated via a Metal-Halogen Exchange Reaction

**Figure 3:**
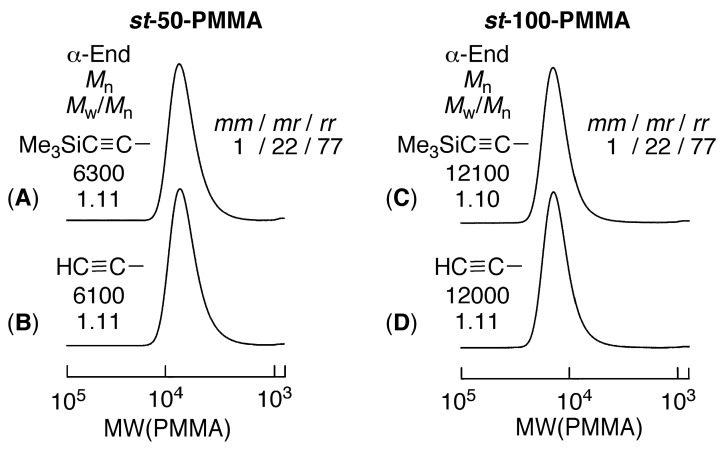
SEC curves of a series of *st*-PMMAs ((**A**,**B**): *st*-50, (**C**,**D**): *st*-100) obtained in DPHLi/**1** in THF at −100 °C and subsequent procedures: (**A**,**B**) Quenching the polymerization ([M]_0_/[DPHLi]_0_/[1]_0_ = 924/34/51 or 1870/34/51 mM) with acidic methanol; (**C**,**D**) Deprotection of Me_3_Si-group by *n*-Bu_4_NF in THF at 0 °C ([Me_3_Si–]_0_/[*n*-Bu_4_NF]_0_ = 10/20 mM).

To synthesize the alkyne-capped functionalized *st*-PMMA, syndiotactic living anionic polymerization of MMA was initiated via a metal-halogen exchange reaction between 1,1-diphenylhexyl lithium (DPHLi) and an α-bromoisobutyric acid ester bearing a Me_3_Si-protected alkyne (**1**) in THF at −100 °C [36]. The 50- and 100-mer alkyne-end-functionalized *st*-PMMAs were synthesized by changing the feed ratios of the monomer to the initiator. The polymers that were obtained after quenching the reaction with acidic methanol showed controlled molecular weights, narrow MWDs (Figure 3A,C), and syndiotactic-rich enchainments (*mm*/*mr*/*rr* = 1/22/77).

**Figure 4:**
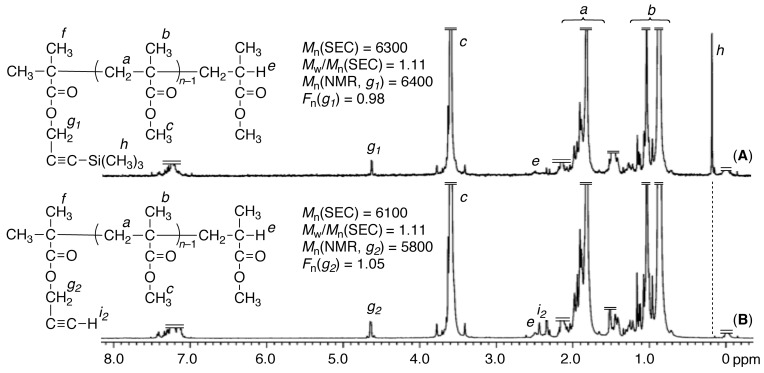
^1^H NMR spectra (in CDCl_3_ at 55 °C) of *st*-50-PMMAs obtained in anionic polymerization of MMA with DPHLi/**1** in THF at −100 °C and subsequent procedures: (**A**) Quenching the polymerization ([M]_0_/[DPHLi]_0_/[1]_0_ = 924/34/51) with acidic methanol; (**B**) Deprotection of Me_3_Si-group by *n*-Bu_4_NF in THF at 0 °C ([Me_3_Si–]_0_/[*n*-Bu_4_NF]_0_ = 10/20 mM).

The ^1^H NMR showed the presence of protected Me_3_Si– (*h*) and methylene protons (*g*_1_) adjacent to the alkyne function. The chain-end functionality of the protected alkyne group (*F*_n_(C≡C–SiMe_3_)) was calculated from the ratio of *M*_n_(SEC) to *M*_n_(NMR), which was calculated from the peak intensity ratio of *g*_1_ to the methyl ester protons (*c*) of the repeating MMA units (Figure 4A). They were close to unity for both of the polymers with different molecular weights (*F*_n_ = 0.98 and 1.12 for *M*_n_(SEC) = 6300 and 12100, respectively), indicating that the alkyne-functionalized α-bromoisobutyric acid ester quantitatively initiates the syndiotactic living anionic polymerization via a metal-halogen exchange reaction in the presence of DPHLi to result in the well-defined alkyne-functionalized *st*-PMMA.

The deprotection of the Me_3_Si-group at the α-end was conducted using tetrabutylammonium fluoride (*n*-Bu_4_NF) in THF at 0 °C. There were almost no changes in the SEC curves with narrow MWDs after the deprotection (Figure 3B,D). The complete and successful deprotection was confirmed by the disappearance of the characteristic Me_3_Si-protons (*h*), and the appearance of a new peak that was assignable to the ethynyl proton (*i*_2_). The chain-end functionalities of the deprotected alkyne group (*F*_n_(C≡C–H)) were similarly calculated from the methylene protons (*g*_2_) and were close to unity (*F*_n_ = 1.05 and 1.04 for *M*_n_(SEC) = 6100 and 12000, respectively). These results show that *st*-PMMA with an alkyne group at the α-chain end was successfully obtained by syndiotactic living anionic polymerization of MMA initiated via a metal-halogen exchange reaction between the protected alkyne-functionalized α-bromoester and DPHLi followed by deprotection. 

### 3.3. Synthesis of it-b-st-Stereoblock PMMA via Copper-Catalyzed Azide-Alkyne Cycloaddition and Stereocomplexation

**Figure 5:**
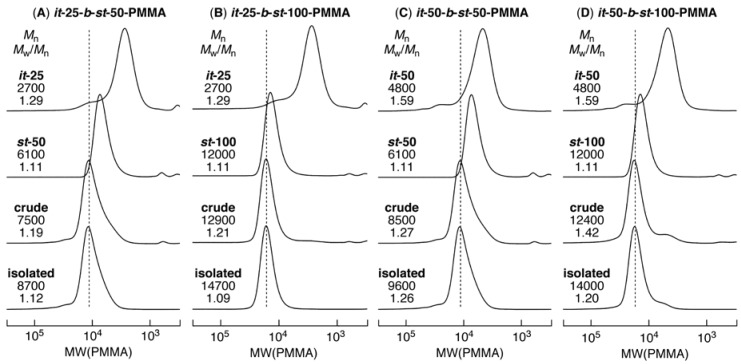
SEC curves of a series of *it*-PMMA-N_3_, *st*-PMMA-C≡CH, and *it*-*b*-*st*-stereoblock PMMAs obtained via CuAAC in the presence of CuSO_4_/sodium ascorbate in DMSO/H_2_O (98/2) at 70 °C ([–N_3_]_0_/[–C≡CH]_0_/[CuSO_4_]_0_/[sodium ascorbate]_0_ = 15/15/5/20 mM) as prepared and after reprecipitations. (**A**) *it*-25-*b*-*st*-50; (**B**) *it*-25-*b*-*st*-100; (**C**) *it*-50-*b*-*st*-50; (**D**) *it*-50-*b*-*st*-100.

Using two series of polymers with different molecular weights, i.e., *it*-PMMA-N_3_ (*DP*_n_ ~ 25 and 50) and *st*-PMMA-C≡CH (*DP*_n_ ~ 50 and 100), CuAAC was conducted with four pairs of the polymers to produce *it*-*b*-*st*-stereoblock PMMAs with different block lengths (*it*-25-*b*-*st*-50, *it*-25-*b*-*st*-100, *it*-50-*b*-*st*-50, and *it*-50-*b*-*st*-100). The click reactions between *it*-PMMA-N_3_ and *st*-PMMA-C≡CH were carried out in the presence of CuSO_4_/sodium ascorbate in DMSO/H_2_O (98/2) at 70 °C, and the two polymers were mixed at the same molar ratio of the two functional groups using each *F*_n_ value. After the reaction, the SEC curves shifted to higher molecular weights (Figure 5A–D) although lower molecular weight peaks slightly remained, most probably due to the presence of a small amount of unfunctionalized prepolymers. The coupling products were further purified by reprecipitation in *n*-hexane to result in narrow SEC curves with higher molecular weights than the starting prepolymers.

**Figure 6:**
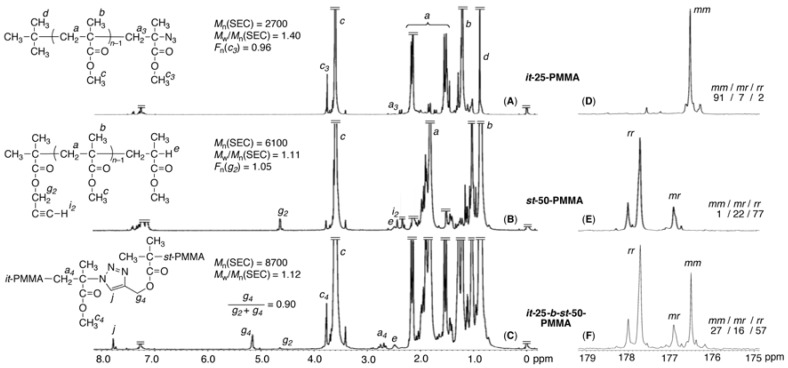
^1^H and ^13^C NMR spectra (in CDCl_3_ at 55 °C) of *it*-25-, *st*-50-, and *it*-25-*b*-*st*-50-PMMAs: (**A**,**D**) *it*-25-PMMA-N_3_ obtained in isotactic anionic polymerization of MMA followed by chlorination and azidation; (**B**,**E**) *st*-50-PMMA-C≡CH obtained in syndiotactic anionic polymerization of MMA followed by deprotection of Me_3_Si-group; (**C**,**F**) *it*-25-*b*-*st*-50-PMMA obtained via CuAAC between *it*-25-PMMA-N_3_ and *st*-50-PMMA-C≡CH in the presence of CuSO_4_/sodium ascorbate in DMSO/H_2_O (98/2) at 70 °C ([–N_3_]_0_/[–C≡CH]_0_/[CuSO_4_]_0_/[sodium ascorbate]_0_ = 15/15/5/20 mM).

In the ^1^H NMR spectrum of the obtained polymers, new peaks for the triazole-ring methine (*j*) at 7.8 ppm and the adjacent methylene (*g*_4_) protons at 5.2 ppm appeared due to the reaction of the alkyne group at the α-end of *st*-PMMA with the azide ω-terminal of *it*-PMMA (Figure 6C). Although a small peak for methylene protons (*g*_2_) was observed due to the unreacted remaining *st*-PMMA prepolymers, the purity of the block polymers was relatively high (90%). The purity was calculated from the peak intensity ratio, *g*_4_/(*g*_2_ + *g*_4_). The ^13^C NMR spectrum of the carbonyl groups of the obtained coupling products (Figure 6F) was a weighed superimposition of the two spectra of *it*-PMMA (Figure 6D) and *st*-PMMA (Figure 6E). The block length ratio was calculated from the tacticities of both the *it*-PMMA and *st*-PMMA prepolymers (*mm*_total_ = *x_it_* × *mm_it_* + *x_st_* × *mm_st_*, *rr*_total_ = *x_it_*× *rr_it_* + *x_st_* × *rr_st_*) and was *x_it_*:*x_st_* = 0.28:0.72, which was close to the theoretical value, *x_it_*:*x_st_* = 0.31:0.69, assuming the formation of *it*-*b*-*st*-stereoblock PMMA with the expected block length. These results indicate that *it*-*b*-*st*-stereoblock PMMAs with various block lengths were efficiently synthesized via CuAAC between the azide-capped *it*-PMMA and alkyne-capped *st*-PMMA (Table 1).

**Figure 7:**
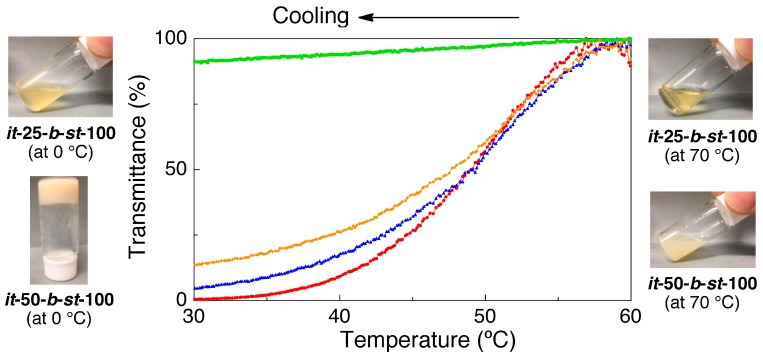
Relative transmittance (wavelength = 500 nm) of a series of SEC curves of solutions of *it*-*b*-*st*-stereoblock PMMAs in CH_3_CN (100 mg/mL) with varying temperatures at cooling rate of 1 °C/min: Orange, *it*-25-*b*-*st*-50; Green, *it*-25-*b*-*st*-100; Blue, *it*-50-*b*-*st*-50; Red, *it*-50-*b*-*st*-100.

The stereocomplexation of these stereoblock polymers was evaluated in CH_3_CN by measuring the transmittance of the solutions (100 mg/mL). Figure 7 plots the relative values as compared to the transmittance at 70 °C. The solutions gradually became turbid as the temperature decreased, and the transmittance of the samples approached 0%, except for *it*-25-*b*-*st*-100. Furthermore, the solution of *it*-50-*b*-*st*-100 formed a gel, whereas no gelation occurred in the other solutions.

According to the most recent studies on stereocomplexation, two *it*-PMMA chains intermolecularly aggregate to form a double helix, which is surrounded by a single helix of *st*-PMMA to form a unique triple helix-like structure [2,7]. In this model, the degree of polymerization of the outer *st*-PMMA chain is double that of the inner *it*-PMMA chain (*it*:*st* = 1:2), while the MMA unit ratio of the total *it*-PMMA chains and *st*-PMMA chain is 1:1 because of the two *it*-chains per *st*-chain in the stereocomplex.

If this model is applied to *it*-*b*-*st*-stereoblock PMMA, the *it*-segments in two block polymer chains can form a double helix to result in a supramolecular structure, e.g., a Y-shape containing two independent single helixes of *st*-segments that are connected to each *it*-segment in the double helix of the *it*-segments. The *it*-double helix segment in the Y-shape supramolecule can be incorporated into a single *st*-helix of another Y-shape molecule to induce further intermolecular associations and finally result in a gel. Among the four *it*-*b*-*st*-stereoblock PMMAs with different block lengths, the *it*-50-*b*-*st*-100 PMMA has the most suitable ratio (*it*:*st* = 1:2) of block lengths for this stereocomplexation model, and can easily result in a gel, whereas the total chain length of *it*-25-*b*-*st*-50 PMMA is shorter and less efficient for gel formation, in spite of the suitable ratio (*it*:*st* = 1:2). Irrespective of the longer *st*-segment in *it*-25-*b*-*st*-100 PMMA, the solution was nearly transparent even at low temperatures, suggesting a low degree of association or intramolecular association due to the long *st*-segment in comparison to that with the short *it*-segment (*it*:*st* = 1:4).

To investigate the effects of the block structure on the stereocomplexation, the four precursor non-block polymers, i.e., *it*-25- or *it*-50-PMMA and *st*-50- or *st*-100-PMMA, were mixed together at the same molar ratio in CH_3_CN. All of the solutions became turbid upon decreasing the temperature. However, no gelation occurred for any of the *it*- and *st*-mixtures at 100 mg/mL. Only the mixture of *it*-50- and *st*-100-PMMAs formed a gel at a higher concentration, i.e., 200 mg/mL. These results indicate that connecting the *it*- and *st*-PMMA segments via a covalent linkage in *it*-*b*-*st*-PMMA enhances gelation via stereocomplexation.

To further confirm the stereocomplexation, the polymers were analyzed by DSC. The precursor linear *it*-50- and *st*-100-PMMA showed *T*_g_ around 35 and 110 °C, respectively. The sample of *it*-50-*b*-*st*-100 PMMA, which was obtained by evaporating CH_3_CN from the solution and dried in vacuo, showed melting temperatures at 166 and 185 °C, which were slightly higher than those that were reported to the stereocomplexes of linear *it*- and *st*-PMMAs [33]. These results also support that the *it*-*b*-*st*-steroblock PMMA forms the stereocomplex.

## 4. Conclusions

The isotactic-syndiotactic stereoblock PMMAs were efficiently prepared via CuAAC between end-functionalized isotactic and syndiotactic PMMAs that were obtained by stereospecific living anionic polymerization of MMA in combination with a metal-halogen exchange reaction for initiation and a halogenation reaction for termination. The reversible transformation between the halide and living anionic chain end, which was used for initiation or termination, enables efficient end-functionalization of stereoregular polymethacrylates, which can be further utilized for the construction of structurally controlled polymers and supramolecular architectures.

## Figures and Tables

**Table 1:** Molecular weight and tacticity of prepolymers and *it*-*b*-*st* PMMAs.

Code	*M*_n_ (*it*)	*mm*/*mr*/*rr* (*it*)	*M*_n_ (*st*)	*mm*/*mr*/*rr* (*st*)	*M*_n_ (*it-b-st*)	*M*_w_/*M*_n_ (*it-b-st*)	*mm*/*mr*/*rr* (*it-b-st*)	*it*-/*st*-Calcd	*it*-/*st*-NMR
*it*-25-*b*-*st*-50	2700	91/7/2	6100	1/22/77	8700	1.12	27/16/57	31/69	28/72
*it*-25-*b*-*st*-100	2700	91/7/2	12000	1/22/77	14700	1.09	18/20/62	18/82	19/81
*it*-50-*b*-*st*-50	4800	92/7/1	6100	1/22/77	9600	1.26	43/13/44	44/56	45/55
*it*-50-*b*-*st*-100	4800	92/7/1	12000	1/22/77	14000	1.20	27/16/57	29/71	32/68
